# Inhibition of PC cell-derived growth factor (PCDGF)/granulin-epithelin precursor (GEP) decreased cell proliferation and invasion through downregulation of cyclin D and CDK 4 and inactivation of MMP-2

**DOI:** 10.1186/1471-2407-7-22

**Published:** 2007-01-29

**Authors:** Yulan Liu, Ling Xi, Guoning Liao, Wei Wang, Xun Tian, Beibei Wang, Gang Chen, Zhiqiang Han, Mingfu Wu, Shixuan Wang, Jianfeng Zhou, Gang Xu, Yunping Lu, Ding Ma

**Affiliations:** 1 Cancer Biology Center, Tongji Hospital, Tongji Medical College, Huazhong University of Science and Technology, Wuhan, Hubei 430030, P. R. China

## Abstract

**Background:**

PC cell-derived growth factor (PCDGF), also called epithelin/granulin precursor (GEP), is an 88-kDa secreted glycoprotein with the ability to stimulate cell proliferation in an autocrine fashion. In addition, some studies indicated that PCDGF participated in invasion, metastasis and survival of cancer cells by regulating cell migration, adhesion and proliferation. Yet the effects of PCDGF on proliferation and invasion of ovarian cancer cells in vitro and the mechanisms by which PCDGF mediates biological behaviors of ovarian cancer have rarely been reported. In the present study we investigated whether and how PCDGF/GEP mediated cell proliferation and invasion in ovarian cancer.

**Methods:**

PCDGF/GEP expression level in three human ovarian cancer cell lines of different invasion potential were detected by RT-PCR and western blot. Effects of inhibition of PCDGF expression on cell proliferation and invasion capability were determined by MTT assay and Boyden chamber assay. Expression levels of cyclin D1 and CDK4 and MMP-2 activity were evaluated in a pilot study.

**Results:**

PCDGF mRNA and protein were expressed at a high level in SW626 and A2780 and at a low level in SKOV3. PCDGF expression level correlated well with malignant phenotype including proliferation and invasion in ovarian cancer cell lines. In addition, the proliferation rate and invasion index decreased after inhibition of PCDGF expression by antisense PCDGF cDNA transfection in SW626 and A2780. Furthermore expression of CyclinD1 and CDK4 were downregulated and MMP-2 was inactivated after PCDGF inhibition in the pilot study.

**Conclusion:**

PCDGF played an important role in stimulating proliferation and promoting invasion in ovarian cancer. Inhibition of PCDGF decreased proliferation and invasion capability through downregulation of cyclin D1 and CDK4 and inactivation of MMP-2. PCDGF could serve as a potential therapeutic target in ovarian cancer.

## Background

PC cell-derived growth factor (PCDGF), also called epithelin/granulin precursor (GEP), is an 88-kDa secreted glycoprotein purified from the conditioned medium of the highly malignant mouse teratoma-derived cell line PC for its ability to stimulate proliferation in an autocrine fashion [1]. In teratoma cells, PCDGF expression was shown to be essential for tumorigenicity [2]. High levels of PCDGF expression are found in rapidly proliferating cells, such as skin cells, deep crypts of gastrointestinal tract, and immune cells. On the other hand, low levels of PCDGF expression are found in cells that are not mitotically active, such as muscle and liver cells [3,4]. Overexpression of PCDGF has been linked to the growth and tumorigenicity of human breast carcinomas and to the acquisition of estrogen independence by estrogen receptor-positive breast cancer cells [5-7]. Despite these strong connections with cancer and growth control, PCDGF 's mode of action is not well understood.

In addition, some studies indicated that PCDGF participated in invasion, metastasis and survival of cancer cells by regulating cell migration, adhesion and proliferation [8-10]. In SW-13 adrenal carcinoma cells, the level of PCDGF expression was a major determinant of the intrinsic activity of the mitogen-activated protein kinase, phosphatidylinositol 3'-kinase, and focal adhesion kinase signaling pathways [9]. PCDGF resulted in exogenously stimulated cell growth and sustained cell survival of both ARP-1 and RPMI 8226 cells in a dose- and time-dependent fashion [10].

The role of growth factors in ovarian cancer development and progression is complex and multifactorial. Growth factors identified to date, such as transforming growth factor-β (TGF-β), macrophage colony stimulating factor (m-CSF), and lysophosphatidic acid (LPA) have been shown to regulate ovarian cancer cell growth and survival *in vitro *and *in vivo *[11-14]. Monica BJ et al have reported that PCDGF was overexpressed in invasive epithelial ovarian cancer and was involved in the stimulation of ovarian cancer cell proliferation [15]. Yet the effects of PCDGF on ovarian cancer in vitro and the mechanisms by which PCDGF mediates ovarian cancer biological behaviors have rarely been reported.

As we know, cyclin D1 can stimulate proliferation by driving cells from the G1 into the S-phase of the mammalian cell cycle. Previous studies suggest that the expression of cyclin D1 could be induced by growth factor stimulation, and cdk4 or cdk6 associated with cyclin D1 exhibits protein kinase activity [16-18]. Matrix metallo-proteinases, a family of zinc-dependent metallo-endopeptidases, are known to be involved in tumor invasion and metastasis by degradation of the extracellular matrix. MMP-2, one of these enzymes, is able to degrade type IV collagen, a major component of the basement membrane [19-21].

Understanding the mechanisms by which PCDGF mediates tumor biological behaviors could be valuable for designing potential therapeutic schemes and improving the survival of ovarian cancer patients. In the present study we investigated PCDGF expression level in ovarian cancer cells. We also observed the proliferation rate and invasion index in Sw626 and A2780 cells after transfection with antisense PCDGF cDNA. The expression of cyclin D1, CDK4 and the activity of MMP2 along with the change of PCDGF were determined.

## Methods

### Cell culture

Human ovarian cancer cell lines SW626, Skov-3 were purchased from the American Type Culture Collection (Manassas). They were maintained in Leibovitz's L-15 and McCoy's 5a media, respectively. Human ovarian cancer cell line A2780 was obtained from China Type Culture Center (Wuhan University) and cultivated in RPMI-1640 medium. All of them were cultured in a 37°C incubator supplied with 5% CO_2 _and 10% fetal bovine serum (Invitrogen).

### Quantitative RT-PCR analysis of PCDGF mRNA expression

Total RNA of three human ovarian cancer cell lines was isolated by TRIzolRNA kit (Gibco BRL) according to the manufacturer's protocol, 5 *μg *of total RNA were reverse transcribed into cDNA, then amplified with equal cDNA. In PCR analysis the specific primer pairs used were for PCDGF: forward primer, 5'-AATGTGACATGGAGGTGAGC-3' and reverse primer, 5'-AGCAGGTCTGGTTATCATGG-3'; for GAPDH as internal standard: forward primer, 5'-CCTTCACCATCTTCCAGGAG-3', and reverse primer, 5'-CCTGCTTCACCACCTTCTTG-3'. The reaction mixtures were subjected to 30 cycles of PCR. Each cycle consisted of denaturation at 94°C for 30 sec, annealing at 58°C for 60 sec and extension at 72°C for 60 sec. 10 μl of each PCR product were analyzed by electrophoresis on 1.5% agarose gels containing 0.5% ethidium bromide.

### Immunoprecipitation and Western Blot analyses

Since PCDGF is a secreted protein, its expression was measured in cell lysates and conditioned media collected in the presence of a protease inhibitor mixture of 200 μM phenylmethylsulfonyl fluoride (PMSF)/1 μM leupeptin/0.5 μM aprotinin/1 mM EDTA (all obtained from Sigma). Cells were lysed in PBS containing 1% Triton X-100 followed by sonication and centrifugation. For comparative studies of PCDGF expression, the samples used for immunoprecipitation and Western blot analysis were normalized to equivalent cell numbers (see figure legends), determined by counting cells from duplicate sets of dishes. Immunoprecipitation of PCDGF was carried out by incubating samples for 4 hr with 5 μg of affinity-purified anti- PCDGF IgG conjugated to agarose beads followed by centrifugation at 10,000 × *g *for 10 min. Immune complexes were resuspended in Laemmli sample buffer, boiled for 5 min, and separated by electrophoresis on a 10% polyacrylamide gel in the presence of SDS. Proteins were electrophoretically transferred to nitrocellulose membrane (Schleicher &Schuell) and conjugated to horseradish peroxidase in the presence of 1% BSA. Immunoreactivity was visualized by the enhanced chemiluminescence detection system (Amersham). The membranes were then blocked with 5% nonfat milk overnight at 4°C and then incubated for 1 hr at room temperature with 10 ng/ml of polyclonal rabbit anti-PCDGF antibody (1:500, Santa Cruz, CA), followed by incubation with HRP-conjugated goat anti-rabbit-IgG. Immunoreactive proteins were detected by an enhanced chemiluminescence kit (Pierce, Rockford, IL).

Quantitative analysis of the RT-PCR and Western blotting was performed with an imaging densitometer.

### Proliferation assay

Ovarian cancer cells in logarithmic growing phase were detached with 0.25% trypsin. Then 5 × 10^5 ^cells were seeded in each well of 24-well plates. After incubation for 72 h, cell monolayers were fixed and stained with 0.5% crystal violet in 20% methanol. Specially bound dye was eluted with a 1:1 solution of 0.1 M sodium citrate (PH 4.2): 100% ethanol. Absorbance at 540 nm was determined using a microplate reader (BTX).

### Matrigel invasion assay

Cell invasion was assessed by a Matrigel invasion assay, using Transwell chambers (Costar) with 5-μm pore polycarbonate filters. The filters were coated with 50 μg of growth factor-reduced Matrigel (VWR Canlab, Montreal, Quebec, Canada). Cells (5 × 10^3^) in 25 μl of serum-free DMEM were placed on each filter, and 26.5 μl of NIH3T3 conditioned medium were placed in the lower chamber as chemo-attractant. 10 h later the filters were washed, fixed, and stained as described. Cells on the upper surface of the filters were removed with cotton swabs. Cells that had invaded to the lower surface of the filter were counted by microscopy selecting 10 random fields per filter (×400 magnification).

### Construction and transfection of antisense PCDGF cDNA expression vector

An antisense PCDGF cDNA expression vector was constructed by inserting a partial human PCDGF cDNA (-30 bp to 465 bp) into a pcDNA3.1(+) mammalian expression vector (Invitrogen) in the antisense orientation. The recombinant plasmid was certified by DNA sequencing. 4 μg of the antisense PCDGF cDNA construct were transfected into SW626 and A2780 cells with Lipofectamine (GIBCO) respectively. Each transfectant was selected and maintained in 800 μg/ml G418, stable clones were isolated after 3 wks and identified by quantitative RT-PCR and Western blot analysis. SW626 and A2780 cells were transfected with pcDNA3.1 (+) empty vector plasmid DNA (vector group). Cells receiving only added Lipofectamine (Lipofectamine group), or only added medium (blank group) were used as controls.

In order to rule out the possibility of the non-specific effect, we also transfected PCDGF siRNA (h) (Santa Cruz, CA) into SW626 and A2780 cells according the manufacture instructions. 48 h after transfection RT-PCR and western blot were used to evaluate the knock down effects.

### MTT assay

Transfected SW626 and A2780 cells were detached with 0.25% trypsin during logarithmic growing phase. After the cell density was adjusted to 10^5^/ml, 100 μl of cell suspension was seeded in 96-well plates, and cultured in corresponding medium containing 10% serum for 48 h. Then 15 μl of thiazolyl blue (10 g/L) was added in each well and incubated at 37°C for 3 h until formazan was formed. Then 100 μl DMSO was added into each well as a solvent. Absorbance was examined at 570 nm using a microplate reader (BTX).

### Western Blot detection of cyclin D1 and CDK4 expression

Total cell lysates were prepared from subconfluent cells using lysate buffer [50 mM Tris HCl (pH8.0), 150 mM NaCl, 0.1% SDS, 100 μg/ml PMSF, 10 μg/ml Aroptinin, 1%NP-40, 0.5%Na_3_VO_4_]. Sixty micrograms of cell lysate were separated by SDS/PAGE on a 10% polyacrylamide gel and transferred to nitrocellulose membrane (Schleicher &Schuell). The membranes were then blocked with 5% nonfat milk overnight at 4°C and then incubated for 1 hr at room temperature with monoclonal mouse anti-cyclin D1 antibody (1:500, Santa Cruz, CA) and polyclonal rabbit anti-CDK4 antibody (1:500, Santa Cruz, CA) respectively, followed by incubation with HRP-conjugated goat anti-mouse-IgG or goat anti-rabbit-IgG. Immunoreactive proteins were detected by an enhanced chemiluminescence kit (Pierce, Rockford, IL).

### Quantitative RT-PCR and zymography analysis of MMP-2 mRNA expression and activity

Total RNA from four groups of human ovarian cancer cells was isolated by TRIzolRNA kit (Gibco, BRL, Gaithersburg, MD) according to the manufacturer's protocol, 5 *μg *of total RNA were reverse transcribed into cDNA, then amplified with equal cDNA. In PCR analysis the specific primer pairs used for MMP-2 were: forward primer, 5'--GTGCTGAAGGACACACTAAAGAAGA--3' and reverse primer, 5'-TTGCCATCCTTCTCAAAGTTGTAGG-3'; Thirty cycles were completed at 94°C for 30s, 58°C for 60s and 72°C for 60s during each cycle.

Gelatin zymography was performed to measure the activity of MMP-2 in SW626 cells. Conditioned medium from equal numbers of cells grown under serum-free conditions for 24 hr were electrophoresed on 10% denaturing SDS/PAGE containing 1 mg/ml gelatin at 4°C, then washed 1 h in 2.5% TritonX-100 and incubated 16 h at 37°C in reaction buffer [50 mmol/L Tris, 10 mmol/L CaCl2, 200 mmol/L NaCl, 1 μmol/L ZnCl2 (pH = 7.5)]. Gels were stained with coomassie blue and destained. Gelatin activities were evaluated by luminance and area of bands against blue background.

### Statistical analysis

All experiments were repeated independently three times, and data were expressed as mean ± SD. Correlation and One-Way ANOVA were used for statistical analysis. *P *< 0.05 was considered as the level of significance.

## Results

### PCDGF mRNA and protein expression

**Figure 1:**
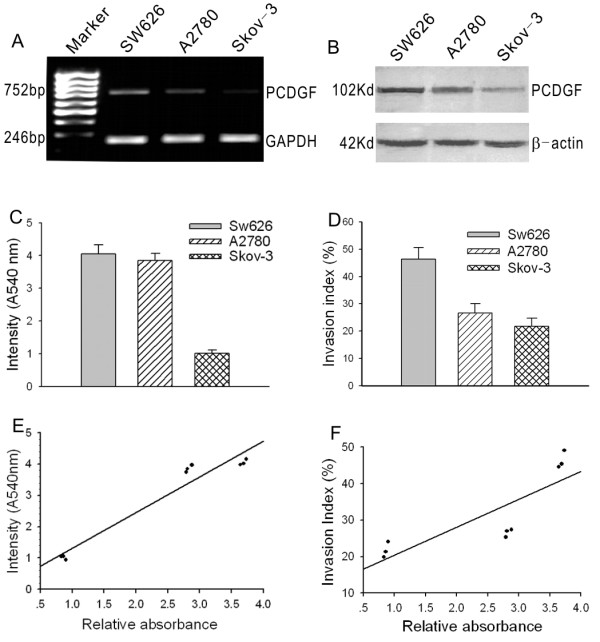
**PCDGF expression in three ovarian cancer cell lines and its correlation with their malignant phenotype**. **A**, PCDGF mRNA expression levels in SW626, A2780 and SKOV3 cell lines were determined by RT-PCR. The figure shows a representative result of three independent experiments. **B**, PCDGF protein expression levels in SW626, A2780 and SKOV3 cell lines were determined by western blot. Also the figure shows a representative result of three independent experiments. **C**, cell proliferation capability of SW626, A2780 and SKOV3 cells determined by MTT assay. Intensity on 540 nm reflects the cell proliferation capability. Values represent the mean ± SE from three independent experiments. **D**, cell invasion capability of SW626, A2780 and SKOV3 cells detected by transwell chambers. The ratio of the number of cells migrating through matrigel to the number of cells migrating without matrigel was defined as invasion index. Values represent the mean ± SE from three independent experiments. **E**, the correlation analysis of PCDGF mRNA expression level and cell proliferation capability using Bivariate Correlations in SPSS12.0. **F**, the correlation analysis of PCDGF mRNA expression level and cell invasion capability using Bivariate Correlations in SPSS12.0.

To detect mRNA and protein expression level of PCDGF in three ovarian cancer cell lines, quantitative RT-PCR and Western blot assay were used. The absorbency ratio of objective bands to reference bands was taken as the quantification standard. In RT-PCR experiments, comparative absorbencies of SW626, A2780 and Skov-3 cells were 3.69 ± 0.05, 2.83 ± 0.05 and 0.86 ± 0.02 respectively (Fig. 1A). One-Way ANOVA analysis exhibited that there are significant differences among groups (*P *< 0.05); In Western blot assay the ratios in the three cell lines were 0.33 ± 0.05, 0.27 ± 0.04, 0.15 ± 0.04 (Fig. 1B). The difference between SW626 and A2780 was not significant (*P *> 0.05), while the differences between either of them and Skov-3 were significant (*P *< 0.05). These results indicate that levels of PCDGF transcription and translation were coincident in three ovarian cancer cell lines

### Cell proliferation and invasion capabilitiy

To investigate the correlation between the expression levels and the malignant phenotype, cell growth assays and Boyden chamber in vitro invasion assays were used to evaluate the capability of proliferation and invasion in these cells. Cell proliferation capability was figured as absorbency at 570 nm. The OD570 in SW626, A2780 and Skov-3 cells were 4.05 ± 0.09, 3.85 ± 0.12 and 1.01 ± 0.06 respectively (Fig. 1C). There were no significant differences between SW626 and A2780 (*P *> 0.05), but there were significant differences between them and Skov-3 (*P *< 0.05).

The ratio of the number of cells migrating through matrigel to the number of cells migrating without matrigel was defined as the invasion index. The invasion index was highest in SW626 (46.34 ± 2.40%), lower in A2780 (26.56 ± 1.09%) and lowest in Skov-3 cells (21.77 ± 2.18%) (Fig. 1D). One-Way ANOVA analysis demonstrated that there were significant differences between SW626 and A2780 or Skov-3(*P *< 0.05), but there was no significant difference between A2780 and Skov-3 cells (*P *> 0.05).

The data indicated that there was a strong correlation between the levels of PCDGF mRNA expression and the capability of proliferation and invasion in ovarian cancer cell (*r*_1 _= 0.97, *r*_2 _= 0.89) (Fig. 1E), GEP protein also reflected this correlation (*r*_1 _= 0.85, *r*_2 _= 0.84) (Fig 1F). Our results suggested that PCDGF may play multiple independent roles in the processes of ovarian cancer occurrence and development.

### Transfection of antisense PCDGF cDNA expression vector

**Figure 2:**
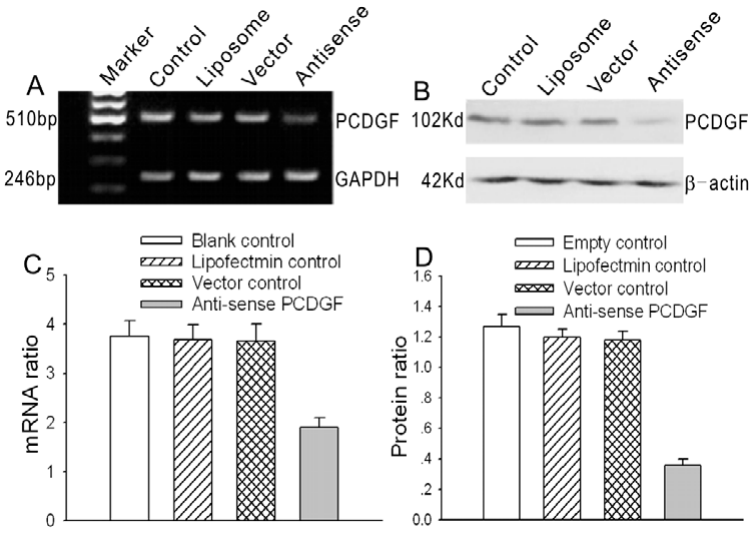
**PCDGF expression level after PCDGF antisense vector and control vectors transfection in SW626 cells**. **A**, PCDGF mRNA expression levels were detected by RT-PCR after transfection of antisense PCDGF construct and three control groups including empty control, liposome control and control vector. The figure shows a representative result of three independent experiments. **B**, PCDGF protein expression levels were detected by western blot after transfection of antisense PCDGF construct and three control groups as above. **C**, quantitative detection of PCDGF mRNA level after PCDGF antisense vector and control vectors transfection in SW626 cells compared with GAPDH. Values represent the mean ± SE from three independent experiments. **D**, quantitative detection of PCDGF protein level after PCDGF antisense vector and control vectors transfection in SW626 cells compared with β-actin. Values represent the mean ± SE from three independent experiments.

To provide a good experimental model for further study of the molecular characteristics of PCDGF, after sequencing, the antisense PCDGF eukaryotic expression vector was transfected into highly malignant ovarian cancer cell lines by lipofectamine. The stable transfectants were selected with G418. The expression levels of PCDGF mRNA and protein decreased sharply in antisense PCDGF transfected cells detected by RT-PCR and western blot. There were significant differences between cells transfected with antisense PCDGF construct and empty vector (*P *< 0.05), The inhibitory rates were 53% of mRNA and 72% of protein expression in Sw626 cells (Fig. 2), and 50% and 70% in A2780 cells (data not shown), respectively. However there are no significant differences between the expression levels of control groups (*P *> 0.05).

Similar results were observed in the PCDGF SiRNA transfected ovarian cells. PCDGF protein expression level was inhibited by 82% in SW626 cells and 76% in A2780 cells 48 h after PCDGF SiRNA transfection (data to be published).

### Effects of antisense PCDGF on proliferation and invasion

**Figure 3:**
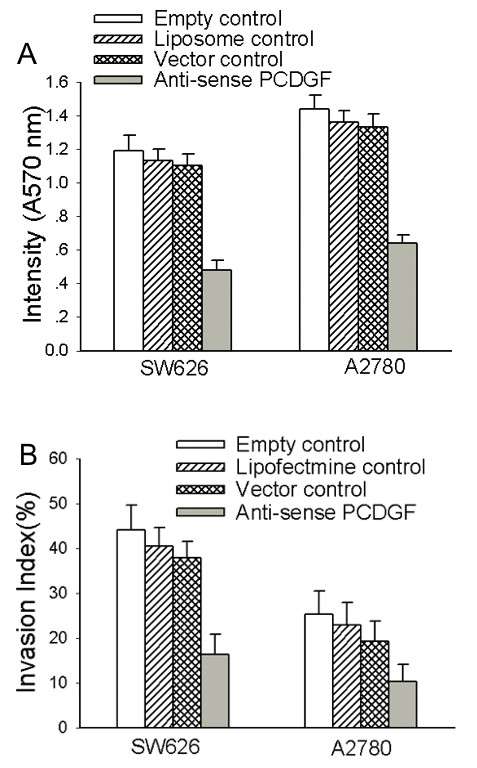
**Cell proliferation and invasion capability decreased after antisense PCDGF construct transfection in SW626 and A2780 cells**. **A**, cell proliferation capability was determined by MTT assay after transfection of antisense PCDGF construct and three control group. Intensity on 540 nm reflects the cell proliferation capability. Values represent the mean ± SE from three independent experiments. **B**, cell invasion capability was detected by transwell chambers. The ratio of the number of cells migrating through matrigel to the number of cells migrating without matrigel was defined as invasion index. Values represent the mean ± SE from three independent experiments. Intensity on 570 nm of cells and invasion index were looked as criterions judging the capability of proliferation and invasion.

To observe the inhibitory effects of antisense PCDGF on proliferation and invasion of highly malignant ovarian cancer cells, MTT assay and Boyden chamber in vitro invasion assay were used to detect the changes of proliferation and invasion capability in those cells before and after transfection. The absorbency on 570 nm and the invasion index decreased almost 2.5 fold in SW626 and A2780 cells transfected with antisense PCDGF construct than with empty vector, with significant differences (*P *< 0.01) (Fig. 3). There were no significant differences between the 3 control groups (*P *> 0.05). These data suggested that antisense PCDGF could remarkably inhibit the proliferation and invasion of highly malignant ovarian cancer cells.

Also similar results were observed in the PCDGF SiRNA transfected ovarian cells. The proliferation rates and invasion index decreased in the SW626 cells and A2780 cells after PCDGF SiRNA transfection (data to be published). These data demonstrated that knockdown of PCDGF by transient transfection of SiRNA could also lead to reversal of malignant phenotype of ovarian cancer cells.

### Expression of MMP-2, Cyclin D1 and CDK4

**Figure 4:**
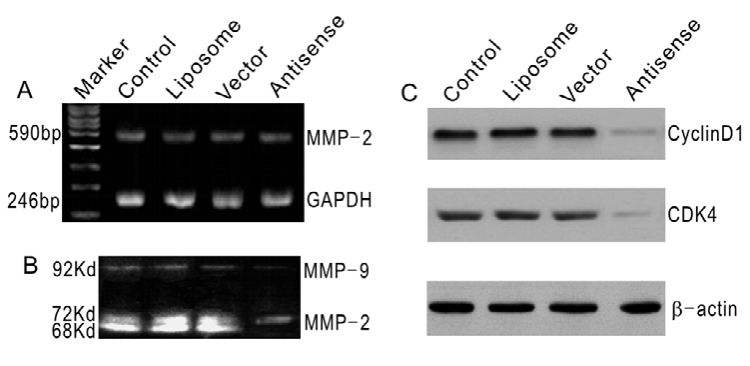
**MMP2, cyclin D1 and CDK4 expression level after antisense PCDGF construct transfection in SW626 cells**. **A**, MMP2 mRNA expression level was unchanged after antisense PCDGF construct transfection in SW626 cells compared with control group detected by RT-PCR. **B**, MMP2 activation was inhibited after antisense PCDGF construct transfection in SW626 cells detected by zymography assay. The 92KD, 72KD and 68KD bands, which represented MMP2 activity, were very weak in antisense PCDGF construct transfection group compared with the control group. **C**, cyclin D1 and CDK4 decreased after antisense PCDGF construct transfection in SW626 cells, which paralleled with the proliferation and invasion capability.

The effects of PCDGF on the expression and activity of MMP-2 were evaluated by a quantitative RT-PCR method and zymography assay respectively. Our results indicated that MMP-2 gene transcription level did not change in SW626 after transfection of the antisense PCDGF, while MMP-9 decreased, which suggested that activation of MMP-2 was inhibited significantly after PCDGF inhibition (Fig. 4A, 4B). To make a pilot study whether other factors were involved in, the protein expression levels of CyclinD1 and CDK4 before and after transfection were detected by Western blot method. These observations provide evidence that the protein expression of CyclinD1 and CDK4 in antisense PCDGF transfected cells was much lower than other transfection groups (Fig. 4C).

## Discussion

Ovarian cancer is the leading cause of death from gynecologic malignancies because of its special characteristics of histology and anatomy [22]. Defining more sensitive biomarkers of ovarian cancer may aid in the development of targeted therapeutics. PCDGF was originally identified through studies of the role of autocrine growth factors on the acquisition of tumorigenic properties in teratoma tumors and purified as a secreted growth factor from the highly tumorigenic teratoma PC cells. Biochemical characterization showed that it was an 88-kDa glycoprotein with a 20-kDa carbohydrate moiety, whereas amino acid sequencing showed that PCDGF corresponded to a family of 6-kDa double cysteine-rich polypeptides. The 6-kDa epithelins, originally purified from rat kidney extracts, were shown to be dual growth modulators for a variety of epithelial cell lines. Recently it was reported that PCDGF might play an important role in tumorigenicity, invasion and survival in various tumors such as human breast cancer, adrenal carcinoma, prostatic adenocarcinoma and multiple myeloma [1-5]. Neutralizing anti- PCDGF antibody reversed basal as well as LPA, ET-1 and 8-CPT-induced ovarian cancer cell growth and induced apoptosis, indicating that PCDGF is a growth and survival factor for ovarian cancer, induced by LPA and ET-1 and cAMP/EPAC through ERK1/2 [23]. However, studies on expression of PCDGF in ovarian cancer cells and the potential molecular basis of its effects on proliferation and invasion have rarely been reported. Few studies indicated a potential mechanism by cyclin and matrix metalloproteinase.

In our study we detected mRNA and protein expression of PCDGF in three ovarian cancer cells by quantitative RT-PCR and Western blot assays. The results indicated that PCDGF was strongly expressed in SW626 and A2780 cells, whereas it was weakly expressed in SKOV3 cells. The expression levels of PCDGF in three ovarian cancer cells were coincident with their malignant phenotype. Cell growth assay and Boyden chamber in vitro invasion assay were employed to evaluate the capability of proliferation and invasion. The capability of proliferation and invasion showed the same trend: SW626 > A2780 > Skov-3. Bivariate correlation analysis indicated that PCDGF mRNA and protein expression were positively correlated with the capability of proliferation and invasion in ovarian cancer. So we hypothesised that PCDGF may play multiple roles in the process of ovarian cancer occurrence and development as an independent factor, and may be a new molecular target for ovarian cancer.

To provide a good experimental model for further study of molecular characteristics of PCDGF, the eukaryotic expression vector for PCDGF antisense RNA was constructed successfully, transfected into highly malignant ovarian cancer cell lines by lipofectamine, and then detected by RT-PCR and Western-blot assay. The expression levels of PCDGF mRNA and protein decreased sharply in antisense PCDGF transfected cells as expected, which suggested that antisense PCDGF might be an effective strategy for specific inhibition of PCDGF transcription and translation in ovarian cancer. Then a good cell model for observing the inhibitory effects of antisense PCDGF on their malignant phenotype of ovarian cancer cells was provided. MTT assay and Boyden chamber in vitro invasion assays were employed to examine the effect of PCDGF on proliferation and invasion the capability. Our results demonstrated that ovarian cancer cells transfected with antisense PCDGF grew more slowly and the invasion capability in vitro decreased sharply, compared with the vector transfected cells. In order to rule out the possibility of the non-specific effect, we adopted SiRNA strategy. The proliferation rates and invasion index were decreased in the SW626 cells and A2780 cells after PCDGF SiRNA transfection as expected.

Interestingly when we introduced antisense PCDGF into less invasive SKov3 cells, cell proliferation rate and invasion index decreased slightly without statistical significance (data not shown), which might indicate that antisense PCDGF worked more efficiently in highly invasive ovarian cancer cells.

Next we investigated the interrelated mechanism of these effects. Cyclin D1, one of the G1 cyclins, was identified independently from human and murine tumors as a putative proto-oncogene associated with chromosomal translocation, gene amplification, and proviral insertion. Constitutive overexpression of cyclin D1 is likely to contribute to the development of malignancy by reducing the dependency on mitogens and other extracellular signals that are normally required to promote progression through the late G1 restriction point [24,25]. In our pilot study the protein expression level of CyclinD1 and CDK4 in antisense PCDGF transfected cells were shown to be much lower than empty vector and other control groups. Our results implied that inhibition of PCDGF decreased cell proliferation and invasion through downregulation of cyclin D and CDK 4.

The changes in the expression and activity of MMP-2 involved in the degradation of the extracellular matrix were evaluated by a quantitative RT-PCR method and zymography assay. As we know, invasion across the basement membrane is among the earliest steps of epithelial tumor progression. Matrix metallo-proteinases, a family of zinc-dependent metallo-endopeptidases, are involved in the degradation of the extracellular matrix, a central element of tumor invasion and metastasis. One of these enzymes, MMP-2, is able to degrade type IV collagen, which is a major component of the basement membrane There was a significant relationship between activated MMP-2 and invasiveness, metastasis, and disease progression [26-29]. In zymography assays we found that the activated band of MMP-2 disappeared in antisense PCDGF transfected cells, suggesting that the antisense PCDGF vector could inhibit the activation of MMP-2 significantly. At the same time it was demonstrated that the antisense PCDGF did not change MMP-2 gene transcription.

In conclusion, our present study demonstrated that PCDGF expression level in ovarian cancer cells correlated well with their potential for proliferation and invasion. Inhibition of PCDGF by antisense PCDGF vector could decrease the proliferation and invasion capability markedly, partially reversing their malignant phenotype. Inhibition of PCDGF decreased cell proliferation and invasion through downregulation of cyclin D and CDK 4 and inactivation of MMP-2. Taken together, PCDGF might be a new target for antisense gene therapy of ovarian cancer.

## Conclusion

Our present study demonstrated that PCDGF expression level in ovarian cancer cells correlated well with their capability of proliferation and invasion capability. Inhibition of PCDGF by antisense PCDGF transfection could downregulate the expression of cyclin D1, CDK4 and MMP-2, and markedly reverses malignant phenotype of ovarian cancer. PCDGF might be a new target for antisense gene therapy of ovarian cancer.

## Abbreviations

PCDGF; PCcell-derivedgrowthfactor, TGF-β; transforminggrowthfactor-β, m-CSF; macrophagecolonystimulatingfactor, LPA; lysophosphatidicacid, PMSF; phenylmethylsulfonyl fluoride, SiRNA; small interference RNA

## Competing interests

The author(s) declare that they have no competing interests.

## Authors' contributions

YL and LX carried out these studies and manuscript preparation. GL, WW, XT, BW, GC, ZH, MW and SW participated in the experiments such as cell culture and preparation of nucleare extracts, flow cytometry analysis, immunofluorescence Staining and extracellular hydrogen peroxide production Assay etc. JZ, GX and YL took care laboratory supplies and maintained laboratory. DM directed the design and performance of the study. All authors read and approved the final manuscript.

## Pre-publication history

The pre-publication history for this paper can be accessed here:
